# BRCA1 and γH2AX as independent prognostic markers in oral squamous cell carcinoma

**DOI:** 10.18632/oncoscience.47

**Published:** 2014-06-01

**Authors:** Joao Paulo Oliveira-Costa, Lucinei Roberto Oliveira, Roberto Zanetti, Juliana Silva Zanetti, Giorgia Gobbi da Silveira, Marcilei Eliza Chavichiolli Buim, Sergio Zucoloto, Alfredo Ribeiro-Silva, Fernando Augusto Soares

**Affiliations:** ^1^ Department of Pathology and Forensic Medicine, Ribeirao Preto Medical School, University of Sao Paulo, Ribeirao Preto, Brazil; ^2^ Department Anatomic Pathology, AC Camargo Cancer Center, Sao Paulo, Brazil; ^3^ Department of Oral Pathology, School of Dentistry, Vale do Rio Verde University (Unincor), Tres Coracoes, Brazil

**Keywords:** BRCA1, H2AX, oral squamous cell carcinoma, DNA damage, prognostic factor

## Abstract

Oral squamous cell carcinomas (OSCC) are believed to originate from sequential mutations that can develop as a consequence of genetic instability acquired over time. BRCA1 are linked to DNA recombination and repair processes, being of importance for its role in regulation of RAD51 and H2AX (γH2AX). The aim of this study was to investigate the relationship between BRCA1 expression status and evaluate its prognostic impact. We selected from 150 OSCC patients, and evaluated BRCA1 expression in OSCC by immunohistochemistry and qRT-PCR, comparing its expression with homologous recombination markers (RAD51, γH2AX and p53), clinicopathological and survival data. Expression of BRCA1 was observed in 61 cases (43.88%) and was related to tumor size (T stage) (p=0.001), and gender (p=0.017). mRNA from *BRCA1* showed a borderline relationship with perineural invasion (p=0.053). BRCA1 [p=0.030; HR: 2.334 (C.I.: 1.087-5.012)], γH2AX [p=0.045; HR: 0.467 (C.I.: 0.222-0.628)] and gender [p=0.001; HR: 10.386 [(C.I.: 2.679-10.623)] were independent prognostic factors for DSS. BRCA1 and γH2AX expression by OSCC cells are associated with reduced overall survival time, independent of other variables in patients, as well as gender, and our findings shed some light about DSB markers in OSCC and its role as prognostic factors.

## INTRODUCTION

Oral squamous cell carcinoma (OSCC) are believed to originate from sequential mutations that can develop as a consequence of progressive genetic instability acquired over time. The identification of reliable factors which can determine a better or worst prognosis is a continuous challenge since the overall mortality rate for OSCC has remained unchanged at approximately 50% over the last several decades, even considering the recent advances in therapies and research [1-3]. Due to the relative unreliability of current prognostic factors, such as TNM staging system, there is considerable interest in discovering prognostic markers that can help to guide efficient therapeutic strategies [1,2,4].

Women with germ-line heterozygous mutations in either BRCA1 or BRCA2 genes has been shown to be at increased risk of developing breast, ovarian and also other cancers [5]. BRCA1 seems to have a broad cellular role, been linked to a wide range of cellular processes such as DNA repair, chromatin remodeling and transcriptional regulation. BRCA1 and BRCA2 are linked to DNA recombination and repair processes, being of particular importance for the role in regulation of RAD51 activity [6,7], which is recruited by a damage signaling protein, phosphorylated H2AX (γH2AX). Cells that lack BRCA1 or BRCA2 have a deficiency in the repair of DNA double-strand breaks by homologous recombination (HR). During the S and G2 phases of cell cycle, DNA repair is done predominately by HR, in a process in which a region of DNA with high sequence identity, usually the identical sister chromatid, is used to copy and replace the damaged DNA sequence. HR is very conservative, potentially error-free, and is a highly reliant process when compared to non-homologous end-joinning (NHEJ) and single-stranded annealing (SSA). RAD51 is a central component to HR, in which it mediates pairing of homologous DNA sequences and strand invasion during the HR process. An inability to repair DSB through HR leads to increased use of the alternative error-prone repair pathways (NHEJ and SSA) with a subsequent increase in deletions, translocations and chromosomal instability. This genomic instability provided by such mechanisms probably underlies the cancer predisposition caused by loss-of-function mutations in BRCA1 or BRCA2 [6,7].

The aim of the present study was to investigate the relationship between the expression of BRCA1 status and various clinicopathologic parameters and immunohistochemical markers (such as p53, γH2AX and RAD51) in two cohorts of oral squamous cell carcinoma samples and to evaluate the prognostic relevance of all variables in terms of disease-specific survival.

## RESULTS

### Epidemiological, clinical and pathological data

A total of 142 patients at our study cohort met the inclusion criteria, and were therefore selected to IHC analysis, although some cases have missing information, such as size, or tobacco and alcohol consumption, and others had no material in TMA slide. Therefore, crossed tables and statistical analysis were based on the number of cases with all the information in each separate analysis. The mean age of the patients in our cohort was 57.89 years, with ages ranging from 32 to 90 years, with a male to female ratio of 7.35:1. A significant percentage of the OSCC patients had reported simultaneous tobacco and alcohol consumption (96 of 131; 73.2%), although for 11 cases, there was no information about smoking and alcohol intake. Regarding histological grading, sixty two patients (43.7%) showed well differentiated tumors, 65 patients (45.8%) showed moderately differentiated tumors, and 13 patients presented poorly differentiated tumors. All demographic and clinical data of the cohort is summarized in Table 1.

**Table 1:** *BRCA1* expression analyzed according to clinicopathologic features in oral squamous cell carcinoma, and homologous recombination markers

Feature		BRCA1+	BRCA1-	p-value
Localization				0.118
	Tongue	23	23	
	Floor of the mouth	19	16	
	Inferior Lip	7	7	
	Palate	17	2	
	Buccal mucosa	3	4	
	Retromolar region	7	6	
	Gingiva	3	3	
Gender				0.017*
	Male	65	59	
	Female	14	2	
Age				0.723
	≤60	52	38	
	>60	27	23	
Smoking				1.000
	Yes	66	49	
	No	9	7	
Alcohol				1.000
	Yes	57	42	
	No	17	13	
				
Size (T stage)				0.001*
	T1/T2	34	48	
	T3/T4	16	3	
Regional Metastasis				0.304
	Yes	38	23	
	No	41	36	
Distant Metastasis				0.138
	Yes	7	1	
	No	72	58	
Tumor histological grade				0.412
	Well	31	30	
	Moderate	40	24	
	Poor	7	6	
γH2AX				0.082
	Positive	51	31	
	Negative	26	30	
RAD51				0.231
	Positive	1	77	
	Negative	4	57	
p53				0.393
	Positive	42	27	
	Negative	37	33	

* Statistical significance determined as p≤0.05

For the 66 cases stored at AC Camargo Cancer Center Biobank the mean age was 62.87 years, with ages ranging between 26 and 92 years. Thirty eight patients were male (66.7%) and 19 were female (33.3%), with a male to female ratio of 2:1. No alcohol or toabacco consumption data were available for this patients. The most common tumor site was tongue, with 21 cases (38.8%), followed by the floor of the mouth and buccal mucosa/gingiva, with 7 cases each (12.96%).

### BRCA1 protein and mRNA expression and clinicopathological data

**Figure 1:**
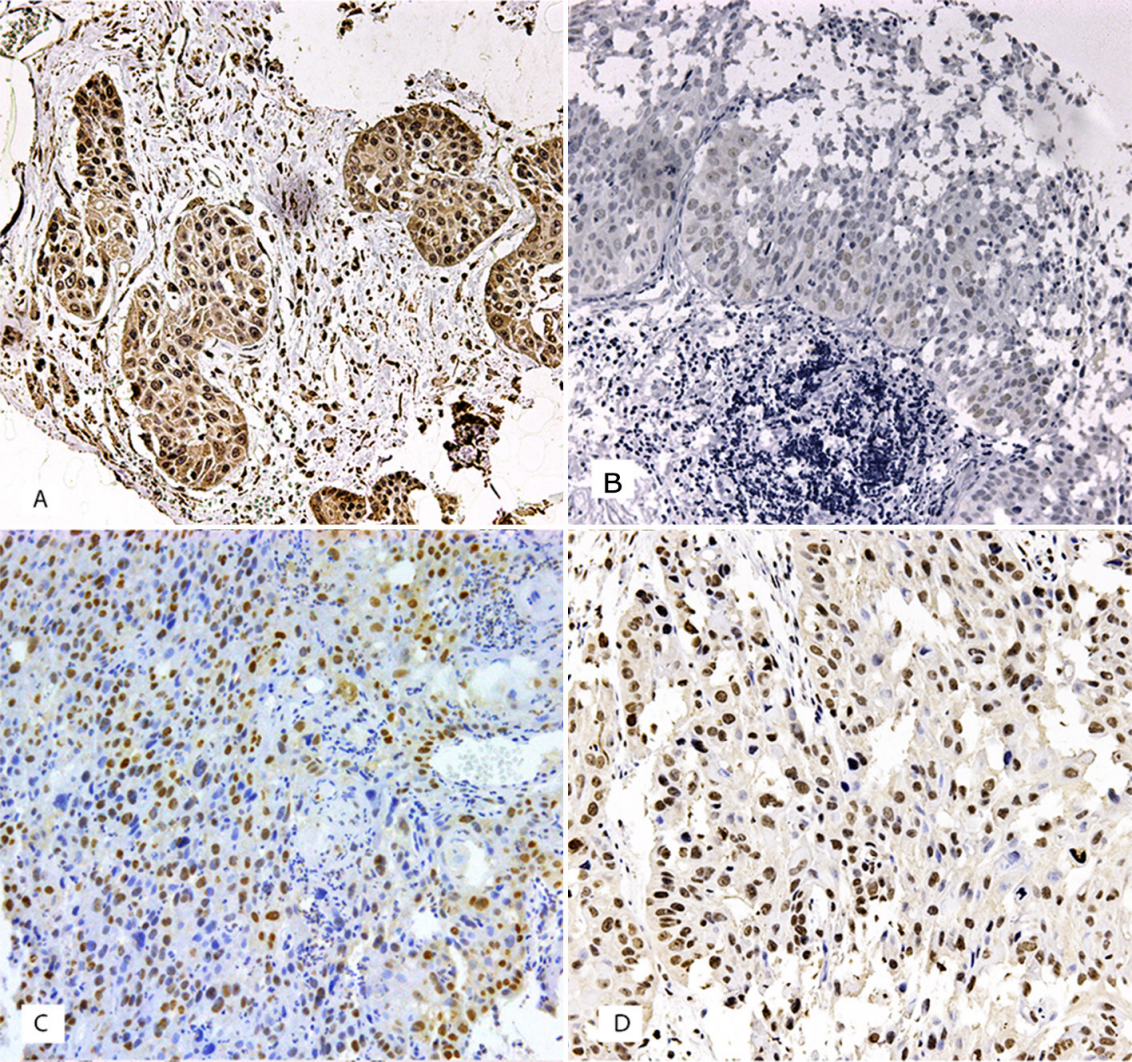
(A) Cytoplasm staining of BRCA1 in neoplastic cells from an oral squamous cell carcinoma (OSCC) (B) Nuclear staining of RAD51 in neoplastic cells from an OSCC. (C) Nuclear staining of p53 in OSCC cells. (D) Nuclear staining of γH2AX in OSCC cells (original magnification ×200 in all photomicrographs).

The cytoplasmatic expression of BRCA1 was observed in 61 cases out of 139 (43.88%). γH2AX expression was observed in 82 cases out of 138 (59.42%), in the nucleus of tumor cells. Nuclear p53 immunoexpression was found in 69 cases out of 139 (49.64%) and nuclear RAD51 expression was observed in 5 cases out of 139 (3.5%). The relationship between BRCA1 expression and clinicopathological data is showed in Table 1. BRCA1, RAD51, p53 and γH2AX expression is showed in Figure 1. We found a relationship between BRCA1 expression and tumor size, with tumor size been grouped as T stage between I/II and III/IV, for statistical purposes (p=0.001), and with gender (p=0.017). There were no significant relationship between BRCA1 expression and localization (p=0.118), alcohol consumption (p=1.000), tobacco consumption (p=1.000), regional metastasis (p=0.304), distant metastasis (p=0.138) and histological grade (p=0.412). BRCA1 expression was not related to γH2AX (p=0.082), RAD51 (p=0.231) or p53 expression (p=0.393).

Regarding BRCA1 mRNA expression, as obtained by RT-PCR reaction, BRCA1 mRNA showed a borderline relationship with perineural invasion (p=0.053), but not with gender (p=0.127), histological grading (p=0.927), angiolymphatic invasion (p=0.286), lymph node metastasis (p=0.303) or clinical staging (p=0.713). RT-PCR results are depicted in Table 2.

**Table 2:** *BRCA1* mRNA expression analyzed according to clinicopathological features of a diferente set of oral squamous cell carcinoma

Feature		BRCA1			p-value
		Low	Normal	High	
Gender					0.127
	Male	9	22	7	
	Female	9	6	3	
Tumor histological grade					0.927
	Well	10	16	5	
	Moderate/Poor(*)	8	12	5	
					
Angiolymphatic Invasion					0.286
	Yes	3	5	4	
	No	15	23	6	
Perineural Invasion					0.053
	Yes	6	14	8	
	No	12	14	2	
Lymph Node					0.303
	Yes	9	13	6	
	No	8	10	1	
Clinical Stage					0.713
	I	2	4	1	
	II	7	8	2	
	III	5	4	2	
	IV	4	12	5	

(*) For statistical purposes, moderate and poor differentiated tumors were grouped.

### Survival analysis

**Figure 2:**
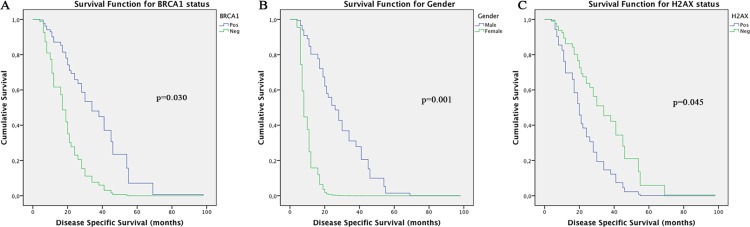
Kaplan-Meier tables generated from Cox Proportional Hazard's analysis showing: (A) BRCA1, (B) Gender and (C)γH2AX impact on OSCC patients survival times

The follow-up period for the OSCC patients in this investigation was used to determine survival rates and ranged from 4 to 108 months (mean = 20 months). The median survival time was 15.5 months. The 5-year disease specific survival rate was 40%. Cox's Proportional Hazards multivariate analysis identified as independent prognostic markers BRCA1 [p=0.030; HR: 2.334 (C.I.: 1.087-5.012)], γH2AX [p=0.045; HR: 0.467 (C.I.: 0.222-0.628)] and gender [p=0.001; HR: 10.386 [(C.I.: 2.679-10.623)], as shown in Table 3. Kaplan-Meier tables are presented in Figure 2.

**Table 3:** Multivariate Analysis in terms of disease-specific survival

Feature		HR	95% Confidende Interval		p-value
Local					
	Tongue	1.000 (Ref)			0.068
	Floor	1.918	0.864	4.259	0.109
	Lip	3.802	1.291	11.201	0.015
	Palate	2.452	0.834	7.207	0.103
	Buccal Mucosa	0.863	0.179	4.155	0.854
	Retromolar Region	0.501	0.120	2.089	0.343
	Gingiva	0.744	0.188	2.942	0.673
BRCA1		2.334	1.087	5.012	0.030*
H2AX		0.467	0.222	0.983	0.045*
RAD51		1.804	0.343	9.497	0.486
p53		0.709	0.374	1.346	0.293
Gender		10.386	2.679	40.265	0.001*
Age		0.883	0.432	1.805	0.734
Smoking		1.656	0.511	5.375	0.401
Alcohol		0.618	0.229	1.668	0.342
Size (T stage)		0.561	0.199	1.580	0.274
Regional Metastasis		1.186	0.572	2.459	0.647
Distant Metastasis		3.041	0.283	32.666	0.358
Tumor Histological Grade	Well	1.000 (Ref)			0.898
	Moderate	0.813	0.922	0.473	0.813
	Poor	0.743	0.198	2.797	0.661

* Statistical significance determined as p≤0.05

Ref: Reference

HR: Hazard Ratio

## DISCUSSION

To the best of our knowledge, the present study is the first study to report the immunoexpression of BRCA1 and γH2AX in OSCC, and to show an important role for these markers as independent predictors of disease-specific survival. There was no influence in disease-free survival for the expression of both markers.

BRCA1, although first thought to be part of a unique multi-subunit complex known as BRCA1-associated genome surveillance complex (BASC), have been implicated in a vast and rising number of different complexes, with distinct functions. These complexes include participation in processes such as cell cycle checkpoint, activation, transcription regulation and DNA repair [6,8,9]. From this processes, BRCA1 role in DNA repair have gained special attention, as it might explains better the BRCA1 role in tumor suppression. In homologous recombination repair, upon DNA damage, H2AX protein is phosphorylated by ATM, and the levels of H2AX phosphorylation is positively correlated with the gradation of DNA damage [10]. Then, γH2AX recruits repair proteins, specially BRCA1 and RAD51, in order to activate HR machinery and DNA damage resolution.

BRCA1 expression has been previously associated with several tumor, such as breast, esophageal, gastric, ovarian, bladder and non-small cell lung tumors. Chen et al. showed that BRCA1 immunoexpression could be affected in tumor cells, due to the observation that in 17 of 17 samples of cells obtained from malignant effusions BRCA1 were found to be expressed in cytoplasm, rather than in the nucleus of these cells [11].

In agreement with recent findings in other tumors such as epithelial ovarian cancer (EOC), gastric, nasopharyngeal carcinoma, [12,13,14] we also found a relationship between BRCA1 expression and different times of survival in our cohort. Lesnock et al. have shown an improved survival in EOC patients with aberrant BRCA1 expression (less than 10% of BRCA1 staining) that have received intraperitoneal cisplatin an paclitaxel. Although the authors report cytoplasmic staining of BRCA1, it is not clear why this staining pattern were not evaluated. Carster et al. reported a different relationship between BRCA1 expression and EOC. Absent/low BRCA1 expression (less than 10% of cell nucleus) was related with a better survival time in patients receiving either platinum treatment, or a combination of platinum and taxane [13]. In OSCC, our findings point to a similar portrait as presented by Lesnock et al., with a higher expression of BRCA1 being a factor of worst disease-specific survival, independently of any other variables [12]. It is important to notice that our cohort were not divided by treatment, as we could not access treatment option for all patients. Notwithstanding, changes in treatment guidelines through the period covered by our research (from 1991 to 2008) would negatively affect the comparison between BRCA1 expression and treatment modalities.

Regarding γH2AX expression, recent reports have shown a growing interest in its prognostic impact in terms of survival. In a cohort of 96 patients presenting non-small cell lung cancer, Matthaios et al. have showed γH2AX as an independent prognostic factor, with a 2.15 fold increase in risk of death in individuals with high expression of γH2AX [15]. In breast tumors, γH2AX overexpression was shown to be a significantly marker of shorter disease-free survival in triple-negative tumors [16]. In endometrial carcinomas, both p53 and γH2AX were analyzed in type I and II tumors, and in univariate analysis, both markers were associated with a shortened disease-free and overall survival [17]. In low-grade bladder urothelial carcinoma, γH2AX expression showed a relationship with recurrence, with negative samples being more likely to show recurrence than positive samples [18].

Recently, γH2AX expression was also reported in circulating tumor cells from patients with breast cancer, both as nuclear foci and peripheral diffuse expression [19]. Its use has been proposed as an important marker of DSB that could provide further insights regarding treatment efficacy directly from blood samples. Although γH2AX has gained attention in other types of tumor, in OSCC its importance has been more described as a peripheral blood marker of radiation schemes, far more than its on-site effects in primary tumor [20,21].

One of the first evidences that BRCA1 could be linked with DNA repair machinery was the cytological observation that, after DNA induced damage, BRCA1 was located at damage-induced foci, together with several repair proteins, such as RAD51 and γH2AX [22]. The DNA damage foci is rapidly marked by the histone H2AX, phosphorylated on serine 139 (γH2AX), and is essential for the accumulation of DNA repair factors, such as BRCA1 and RAD51. As BRCA1 has E3 ubiquitin ligase activity, it is believed BRCA1 could mediate ubiquitylation at DNA damage sites, facilitating the DNA damage response [23]. Although it seems a clear mechanism by which BRCA1 deficiency would cause a impaired resolution of DNA damage, there are evidences that BRCA1 impairment may not affect cell proliferation and survival at all times. *Brca1*-null embryonic stem cells from mice, and showing expression of a BRCA1-mutant (BRCA1-S988A) displayed no differences in cell cycle, or sensitivity to DNA damaging agents when compared with BRCA1-wild cells.

As stated, during DNA damage, BRCA1 expression was supposed to be at nucleus of cancer cells, at the site of the DNA damage. Our results points to a cytoplasmic expression of BRCA1, rather than nuclear. Although far from the scope of our article, is important to note that Chen et al. have reported for the first time the aberrant localization of BRCA1 in breast cancer cells cytoplasm, pointing that the sub cellular localization of BRCA1 suggests that abnormalities in BRCA1 are fundamental in breast tumors, and could be caused by intragenic mutation, and so on, loss of function of BRCA1 [11]. Also our group have reported recently BRCA1 cytoplasmic expression in invasive breast ductal carcinomas [24]

In conclusion, both BRCA1 and γH2AX expression by OSCC cells are associated with reduced overall survival time, independent of other variables in patients. Although further studies are needed to better understand BRCA1 and γH2AX role in OSCC development, our findings shed some light about DSB markers in primary tumors, and can be further extended with more DSB molecules in future, in order to allow a better understanding of DNA repiclational machinery status in OSCC development, and also its prognostic impact in OSCC.

## MATERIALS AND METHODS

### Ethics Statement

The present study was approved by the Institutional Ethics Review Board of Ribeirao Preto Medical School (number 2401/2008) and by the Institunional Ethics Review Board of AC Camargo Cancer Center (number 1416/2010), following both institutional and national guidelines. All written consents were obtained from all patients for the use of biological material, as well as for the use of their information, and storage at hospital database. When written consent was not possible to be obtained, according to national guidelines, the reasons for not doing so were provided to Institutional Ethics Review Board in order to obtain authorization for the use of samples.

### Case Selection

A total of 150 cases of primary OSCCs diagnosed between 1990 and 2009 were retrieved from the medical files of the General Hospital of Ribeirao Preto Medical School, University of Sao Paulo, Brazil. The files were reviewed and analyzed to collect information regarding age, gender, smoking and alcohol intake history, primary tumor site, histological classification, treatment, tumor recurrences, regional and distal metastasis, disease-free survival (DFS) and overall survival (OS) of the patients.

The inclusion criteria for this study were as follows: 1) adequate clinicopathological data with sufficient follow-up (at least 2 months); 2) the availability of sufficient paraffin embedded tumor material; 3) the presence of oral cavity cancer (including oral tongue, floor of the mouth, gingiva, lips, buccal mucosa, hard palate, and retromolar trigone) (ICD-10: C00, C02-C06) [25]; 4) no history of previous head and neck cancer; 5) no previous radio-or chemotherapy; and 6) the absence of distant metastasis. Patients with in situ tumors were excluded from the study, as well as those who died of other unrelated causes.

The patients were dichotomized by tobacco and alcohol consumption into “never consumer” and “current consumer” groups, according to previously described standardized criteria [26]. Similarly, tumor recurrence was defined as the occurrence of another carcinoma ≤ 2 cm away from the primary carcinoma [27] and the tumors were classified as either well-, moderately, or poorly differentiated according to the World Health Organization (WHO) histological differentiation grade classification [28]. Disease-specific survival was considered as deaths due only to cancer, with other causes of death being considered as censored data. For each case, all available hematoxylin and eosin (HE)-stained sections were reviewed to confirm the diagnosis of OSCC and to select a representative tumor area for Tissue Microarray (TMA) construction and immunostaining.

For qRT-PCR, 66 cases were retrieved from AC Camargo Cancer Center Biobank, and only pathologycal information was retrieved. The same selection criteria used to FFPE samples was also applied to the selected samples.

### TMA Construction

For each case, all available HE-stained sections were reviewed to confirm the diagnosis of OSCC and to delineate the most significant tumor region in each case for inclusion in the construction of a TMA paraffin block. Two tissue cylinders of each OSCC case (diameter, 1 mm) were punched from the selected regions of each of the 150 donor paraffin blocks and arrayed into a new recipient paraffin block using a Manual Tissue Arrayer I (Beecher Instruments, Silver Spring, USA). Three-micron-thick sections were cut from the TMA paraffin block using the Paraffin Tape-Transfer System (Instrumedics, Saint Louis, USA). One section was stained with HE to confirm the presence of the tumor, and the other sections were subjected to immunohistochemical (IHC) analysis.

### Immunohistochemistry

All of the tissue samples were fixed in 4% neutral formalin and embedded in paraffin. The IHC staining was performed using the Dako ENVISION system (Dako, Carpinteria, CA, USA). The sections were de-paraffinized in xylene and rehydrated through a series of graded alcohols. The endogenous peroxidase activity was blocked for 30 minutes in a solution containing 0.3% hydrogen peroxide. Antigen retrieval was performed by incubating the sections in a 10-mM citrate buffer for 40 minutes in a vapor lock. After antigen retrieval, the specimens were allowed to cool for 30 minutes and were then incubated at 4ºC overnight with the indicated primary antibodies. The dilutions and sources of the primary antibodies used in this study were as follows: BRCA1 (1:100, clone MS-13, Serotec, Oxford, UK), γH2AX (1:100, clone 3F2, Abcam, Cambridge, USA), p53 (1:100, clone DO-7, Novocastra, Newcastle upon Tyne, UK) and RAD51 (1:50, clone 51RAD01, Abcam, Cambridge, USA). After an overnight incubation with the primary antibody, the slides were incubated with post-primary solution for 30 minutes and were then incubated with the polymer for 30 minutes (both provided by the Dako ENVISION system). The reactions were developed with diaminobenzidine (DAB), followed by Mayer's hematoxylin counterstaining. The slides were then dehydrated in a graded series of ethanol and mounted with Permount (Fischer, Fairlawn, NJ).

Neoplastic cells were examined for cell cytoplasmic immunostaining of BRCA1. As no cut-off values were available for oral squamous cell carcinoma, we chose to use the most recent cut-off used in a large cohort of ovarian cancer patients, and a sample were considered positive when more than 10% of the cells showed cytoplasmic staining [12]. The specimens were defined as γH2AX-positive if more than 10% of the nucleus in tumor cells were distinctly immunostained [29]. For RAD51, as we found a very weak staining, samples with cells presenting nuclear staining were considered positive. For p53, tumors with more than 10% of stained nucleus were considered positive [26]. All immunohistochemistry evaluation and analysis were carried out blinded to the patients' clinical outcomes.

### RNA extraction

Approximately 30 mg of frozen tissue was homogenized with Precellys 24® equipment (Carlsbad, California, USA). Afterward, the supernatant was used to purify total RNA with the RNeasy Mini kit (Qiagen, Venlo, the Netherlands) according to the manufacturer's protocol. The quantity and purity of RNA samples were assessed with NanoDrop^™^ ND-1000 (Thermo Scientific, Wilmington, Delaware, USA). RNA integrity was controlled with the Agilent Bioanalyzer 2100 (Agilent Technologies, Palo Alto, California, USA). Only RNA samples with OD 260/280 >1.8 and RIN (RNA Integrity Number) >5 were further processed.

### qRT-PCR

Total RNA was extracted from frozen tumor specimens as previously described (RNA extraction section) and then reverse transcribed using High Capacity cDNA Reverse Transcription Kit with RNase Inhibitor (Invitrogen, Carlsbad, CA, USA) according to the manufacturer's instructions. The probe used in this study was BRCA1 (catalog number Hs01556193_m1).

RT-PCR was performed in a total reaction volume of 20 μl, including 10 μl Taqman Universal MasterMix II, 1 μl of TaqMan Probe, 2 μl of cDNA, 7 μl of double-distilled water. The quantitative real-time PCR reaction was set at an initial denaturation step of 10 min at 95º C; and 95ºC (5 seconds), 60ºC (60 seconds). All experiments were performed in duplicate, using Bio-Rad's CFX96 detection system (Bio-Rad, Hercules, CA, USA). All samples were normalized to GAPDH. The median in each duplicater was used to calculate relative mRNAs concentrations (DCt =Ct median target gene - Ct median GAPDH). Expression fold changes were calculated using 2-DDCt methods [30]. Fold changes greater than 2 were considered of high expression, fold chenges lesser than 0.5 were considered of low expression, and fold changes within this range were considered as normal expressed.

### Statistical analysis

The statistical analyses and tests were performed with the commercially available IBM SPSS Statistics 21.0 software (IBM, Chicago, IL). Descriptive statistics were used to summarize the study data. The DFS period was defined as the time from diagnosis until the occurrence of local recurrence or metastasis (the metastases were confirmed through histopathological analysis), and OS period was defined as the interval between the diagnosis and the date of death for the uncensored observations or the date of the last information recorded in the medical records for censored observations. To evaluate the prognostic significance BRCA1 immuexpression and mRNA expression in OSCCs, the data was analyzed together with the clinicopathological features through cross tables using Fisher's exact or chi-square tests. A value of p≤0.05 was considered as statistically significant. A multivariate Cox proportional hazard regression model was used to build models containing a subset of candidate risk factors with independent prognostic properties, and the Kaplan-Meier method to estimate and compare the cumulative survival rates after multivariate analysis. Statistical significance was defined as a two-tailed P-value ≤ 0.05.
